# Association of Park Size, Access and Neighbourhood Walkability with Physical Activity and Obesity: A Cross-Sectional Analysis

**DOI:** 10.3390/ijerph23060787

**Published:** 2026-06-11

**Authors:** Ghazal S. Fazli, Jane Polsky, Ashley Johns, Peter Gozdyra, Jin Luo, Gillian L. Booth

**Affiliations:** 1 Department of Geography, Geomatics, and the Environment, University of Toronto Mississauga, Mississauga, ON L5L 1C6, Canada; 2 ICES, Sunnybrook Health Sciences Centre, Toronto, ON M4N 3M5, Canada; 3 Statistics Canada, Ottawa, ON K1A 0T6, Canada; 4 Map Centre for Urban Health Solutions, Li Ka Shing Knowledge Institute, Unity Health Toronto, Toronto, ON M5B 1X2, Canada; 5 Department of Medicine, University of Toronto, Toronto, ON M5S 3H2, Canada; 6 Institute of Health Policy, Management and Evaluation, University of Toronto, Toronto, ON M5T 3M6, Canada

**Keywords:** neighbourhood walkability, park and greenspace access, physical activity, obesity, urban health, city planning, population health

## Abstract

**Highlights:**

**Public health relevance—How does this work relate to a public health issue?**

- Obesity is a major global health challenge.
- Physical inactivity is a key driver of the rise in obesity in the population.

**Public health significance—Why is this work of significance to public health?**

- There is growing evidence that people who live near parks are more physically active.
- Greenspace interventions have become a key target in public health efforts to promote physical activity.

**Public health implications—What are the key implications or messages for practitioners, policy makers and/or researchers in public health?**

- The presence of parks in highly walkable neighbourhoods was associated with a further increase in both leisure and transport-related physical activity.
- The addition of a park in low-walkability areas appeared to have limited impact on physical activity levels.

**Abstract:**

Background: We examined whether higher access to parks and greenspace is independently associated with an increase in physical activity and lower rates of obesity when neighbourhood walkability is accounted for and whether neighbourhood walkability and park access have synergistic effects on these outcomes. Materials and Methods: We used cross-sectional data from the Canadian Community Health Survey between 2007 and 2014 for adults aged 20 to 74 in Ontario, Canada. Neighbourhood-level park access exposures included size of parks and number of parks within 800 m of residential areas, and neighbourhood walkability was based on a validated index. The main outcomes were physical activity during leisure time (LPA), both leisure and transportation physical activity (LTPA), and obesity. Descriptive and multivariate logistic regression analyses were conducted, stratified by age groups, accounting for sex, income, ethnicity, and season. Results: Among 41,945 respondents, park access was associated with higher LPA and LTPA, with effects modified by neighbourhood walkability (*p* < 0.001). Physical activity was highest in neighbourhoods with high walkability and park access and lowest in low walkability areas without parks. In highly walkable neighbourhoods, ≥1 small- or medium-sized park was associated with 29% higher odds of LPA (OR: 1.29, 95%CI: 1.21–1.37) and 48% higher odds of LTPA (OR: 1.48, 95%CI: 1.38–1.57) than low walkability/no park access. In contrast, associations were modest in low-walkability neighbourhoods (4–7%). High walkability was also associated with lower obesity and marked reductions when combined with very high access to large parks (OR: 0.72, 95%CI: 0.55–0.94). Findings were consistent across age groups. Conclusions: High neighbourhood walkability was the strongest predictor of physical activity and lower obesity risk, with park access providing additional benefits primarily in already walkable environments. These findings suggest that population health interventions targeting urban design need to consider the combined benefits of neighbourhood walkability and park access on health.

## 1. Introduction

There is a growing recognition of the impact of natural environments on health and wellbeing. Research has shown that populations that live nearer to greenspace, including parks, forests, and other natural settings, have lower rates of obesity [1], type 2 diabetes [2], cardiovascular disease [3], mental health disorders [4], and all-cause mortality [5,6], compared to those who live further away from these settings. The potential pathways through which access to greenspace might lead to better health are multifold [7]. Parks and trails provide opportunities for physical activity and social connection [8,9,10] and are associated with improved mental health, stress relief and social capital [7]. Greenspace also mitigates the effects of environmental harms, such as air and noise pollution, and provides protection from extreme heat [11]. Hence, as cities strive to create healthy, livable communities, there has been a growing discourse around the central role of parks and greenspace in urban design. These public spaces are also becoming a key target for population-wide interventions to curb the epidemic of obesity and related chronic diseases [12,13].

A growing body of research suggests that people living in neighbourhoods that have more parks, trails and tree cover are more physically active [14,15,16,17,18,19,20]. However, these studies vary considerably with respect to their study designs and the methods used to measure access to greenspace. While much of this evidence is derived from North American and European settings, other studies from Asian cities such as Bangkok have shown how differences in density, climate, and transportation systems shape park use and physical activity in distinct ways [21,22,23]. Further, the majority of studies on this topic fail to account for other environmental influences, such as neighbourhood walkability and its combined effect with parks and greenspace on physical activity. High neighbourhood walkability is associated with lower rates of obesity and related diseases, including the development of prediabetes, diabetes, and cardiovascular diseases [24,25,26,27,28,29,30]. The health benefits of walkability are due in large part to its impact on walking and other active forms of transportation [30]. Because neighbourhood walkability and park access are not evenly distributed throughout urban areas, communities may have favourable or unfavourable profiles for both characteristics. Historical planning practices have led to widespread changes over time in density and zoning standards, as well as the requirements for greenspace within residential areas. As a result, those living in highly walkable neighbourhoods may have greater access to parks or they may be more likely to use parks simply because of the ease of travelling there. This study is grounded in an ecological model of health behaviour, which posits that physical activity is shaped by interacting environmental influences [31]. Within this framework, walkability may facilitate mobility through walking and access to local destinations, while parks provide destinations for activity, suggesting that their combined presence may produce synergistic effects than either alone. In many cities, affluent neighbourhoods also have substantially more tree coverage and greater park numbers, quality and safety [32,33,34]. This confluence of factors have created a non-equitable pattern of walkability and greenspace in cities, which makes it more challenging to disentangle the benefits of one factor over another. Furthermore, studies on park access and physical activity are not sufficiently large enough to identify subsets within the population who would benefit most from these exposures [35,36].

The objectives of this study were to examine whether having better access to parks and greenspace is independently associated with an increase in physical activity and lower rates of overweight and obesity when neighbourhood walkability is accounted for, and to examine whether neighbourhood walkability and park access have synergistic effects on these outcomes. We hypothesized that residents living in more supportive environments (greater walkability/park access) will report having higher levels of physical activity and lower rates of obesity compared to those living in the least walkable areas with limited or no access to parks. We also hypothesized that these differences would be magnified by the size and number of parks that residents have access to, such that having one or more parks within walking distance or access to larger parks will result in greater levels of physical activity and lower rates of overweight and obesity. Because prior studies have shown that park and greenspace use varies by age [37,38,39], we examined whether any observed associations were greater amongst younger versus older adults.

## 2. Materials and Methods

### 2.1. Study Design and Population

This study analyzed existing data collected from participants in one of eight waves of the Canadian Community Health Survey (CCHS), conducted between 2007 and 2014. The CCHS is a national cross-sectional survey developed by Statistics Canada that collects health-related information from a representative sample of the Canadian population (aged 12 and above) annually. Our analysis included adults aged 20 years and older who lived in one of 15 cities in Southern Ontario at the time they participated in the CCHS, including Hamilton, London, Ottawa, Toronto, and surrounding cities. Survey responses were linked to neighbourhood-level measures using each participant’s neighbourhood of residence at the time of CCHS entry. This research was conducted at ICES, a nonprofit research institution affiliated with the University of Toronto, which holds de-identified and linkable administrative health databases and surveys.

### 2.2. Neighbourhood Measures

Park access: We derived park access variables for all neighbourhoods within our study area. Neighbourhoods were based on dissemination areas (DA), which are small residential units created by Statistics Canada that span one or more adjacent city blocks and include a population of 400–700 residents [40]. A geographic information system (GIS)-based network analysis that considered existing roads and foot paths was used to create an 800 metre (m) buffer around the centre of each DA, corresponding to a distance that could be reached within 10 min of walking. Park access measures were captured using the 2009 CanMap Parks and Recreational polygon data for our study area from DMTI Spatial Inc. (Richmond Hill, ON, Canada) [41]. We included official city parks of all sizes, as well as outdoor recreational spaces (sports fields), provincial parks, parks with conservation areas, parks with trail systems, and cemeteries that were 10 hectares or greater. We excluded areas that were not considered important for recreational and transportation physical activity such as botanical gardens, campgrounds, migratory bird sanctuaries, picnic sites, and hydroelectric power corridors; however, official city parks that reside within these corridors were included. Neighbourhood-level access to parks was defined as low (0 parks), moderate (1–2 parks), and high (≥3 parks) based on the number of parks that were accessible within the 800 m buffer. Because the size of the park determines the type of activities that can be undertaken within its space, we further categorized access based on park size (small or medium: 0.5–4.9 hectares; large parks: ≥5 hectares).

Neighbourhood walkability: DA-level walkability was assigned using a validated index created for our study area, as previously described [24,25,26,27,28,29,30]. Briefly, the index comprises four equally weighted components based on street connectivity, population density, residential density, and number of retail stores, services and other walkable destinations. Standardized walkability scores ranging from 0 to 100 were divided into five quintiles (Q) with an equal number of DAs, with Q1 representing the lowest level of walkability and Q5 the highest level of walkability. In previous research, we observed a threshold effect whereby residents living in the highest quintile had a substantially lower rate of obesity and diabetes incidence compared to the 4 lowest quintiles of walkability [24]. Therefore, for our primary analysis the index was divided into two categories only: low walkability (based on quintiles 1–4) and high walkability (based on quintile 5). This threshold-based approach was used to enhance interpretability and to distinguish environments most supportive of physical activity and related health outcomes.

### 2.3. Individual-Level Measures

Individual-level covariates were ascertained from self-reported responses in the CCHS, including age, sex, ethnicity, and household income. Self-reported ethnicity was categorized as white or non-white only, due to limitations in the number of participants from different ethnoracial groups. Further, we ascertained the season during which each CCHS interview was conducted to account for seasonal changes in physical activity and body weight. These were assigned to participants based on the mean temperature during the 3-month window prior to their interview date. As detailed below, this served as the period in which physical activity was reported. This 3-month period was considered to be summer for interviews conducted between July and October; winter for interviews conducted between January and April; and a temperate season (Spring/Fall) for interviews conducted during the months of May, June, November, or December.

### 2.4. Outcomes

Physical activity was measured using derived variables from the CCHS that reflect how physically active an individual is during both leisure time and while engaged in transportation. The survey collected information from participants regarding the frequency, duration, and type of physical activity undertaken during the three months prior to their interview date. This was used to calculate their total daily energy expenditure in kcal/kg/day, which was then assigned to one of three categories: ‘inactive’ (<1.5 kcal/kg/day), ‘moderately active’ (1.5 to <3 kcal/kg/day), and ‘highly active’ (≥3 kcal/kg/day). In this study, we combined moderately and highly active into a single category of moderate-to-high physical activity versus inactive and reported a combination of leisure and transportation physical activity (LTPA) and leisure physical activity alone (LPA) separately.

Obesity: Ethnic-specific body mass index (BMI) measures were created based on self-reported height and weight from the CCHS. BMI was used to categorize participants as overweight or obese (≥25 kg/m^2^) and obese (≥30 kg/m^2^). For South Asian and Chinese populations only, BMI categories of ≥23 kg/m^2^ and ≥25 kg/m^2^ were considered overweight or obese and obese, respectively, based on categories defined by the World Health Organization and prior studies [42,43].

### 2.5. Analyses

Descriptive statistics were calculated for baseline characteristics of the population, according to park access exposures (≥1 park versus 0 parks within 800 m). Multivariate logistic regression models were used to examine the relationships between park access (0 parks, 1–2 parks, ≥3 parks within 800 m) and each outcome: physical activity during leisure time (LPA), both leisure and transportation physical activity (LTPA), and obesity. Models were stratified by park size (small or medium-sized versus large parks), and age group (20–49 and ≥50). We adjusted for covariates including age (as a continuous measure), sex, household income, ethnicity, season, and neighbourhood walkability.

To determine whether associations between park access, physical activity, and obesity were modified by neighbourhood walkability, we tested for interactions between park access (i.e., ≥1 park, no park) and walkability (high/low) in each model. For those that were significant, we performed stratified analyses to examine the effects of park access on each set of outcomes within high- and low-walkability neighbourhoods. Further, we examined the effects of walkability on each set of outcomes in areas with high and low access to parks. We ran further models with four neighbourhood categories: both high walkability and park access, low walkability and park access and areas with high/low and low/high walkability/park access. To verify that our high/low categorization of walkability was appropriate, we conducted sensitivity analyses in which all 5 quintiles of walkability were used to examine associations with physical activity outcomes and obesity. These too were stratified by high/low access to parks. All analyses were run using SAS Enterprise 9.4.

### 2.6. Ethics Approval

ICES is an independent, nonprofit research institute whose legal status under section 45 of Ontario’s Personal Health Information Protection Act provides the opportunity to collect and analyze deidentified health care and demographic data, without consent or review by a Research Ethics Board, for the purpose of health system evaluation and improvement.

## 3. Results

Overall, there were 41,945 CCHS participants living in our study area between 2007 and 2014, of which 34,075 lived in a neighbourhood that had at least one park within walking distance. Baseline characteristics of the study population are provided in Table 1. The average age of the study population was 51.2 years, 43% were from a high-income household and 63% had post-secondary education. The population characteristics were generally similar in communities that had access to parks and those that did not. As an exception, a higher proportion of those who had access to a park within 800 m of their home self-identified their ethnoracial identity as white compared to those who did not have access (72.2% and 67.2%, respectively). Further, residents who had no park access were more likely to live in a low-walkability neighbourhood.

### 3.1. Park Access, Walkability and Physical Activity

**Figure 1:**
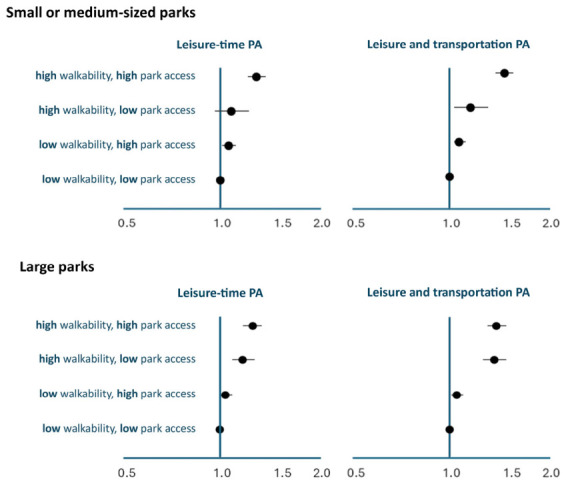
Combined effects of neighbourhood walkability and park access on the odds of being physically active, by park size. PA: physical activity; reference group: low walkability and low park access; high walkability was based on quintile 5 and low walkability was based on quintiles 1–4. High park access was defined as ≥1 parks within 800 m; low park access was defined as 0 parks within 800 m. Adjusted for age, sex, ethnicity, season, household income, neighbourhood walkability, park access, and interaction terms between neighbourhood walkability and park access; data sources: data for LTA, LTPA and obesity were attained from Canadian Community Household Survey (CCHS) cycles 2007–2008, 2009–2010, 2011–2012, and 2013–2014; data for parks were attained from DMTI, Ministry of Education, and City of Toronto.

Overall, there was a significant association between park access and physical activity; however, these effects varied significantly according to neighbourhood walkability (*p* < 0.001 for interaction). Figure 1 examines the combined effects of neighbourhood walkability and park access defined as the presence of one or more parks of a specific size. Overall, physical activity levels related to leisure (LPA) or leisure and transportation (LTPA) were greatest in the most favourable environments (high walkability/moderate to high park access) and lowest in the least favourable environments (low walkability/no park access). In most scenarios, the addition of one or more parks led to a further increase in physical activity levels in both low- and high-walkability settings, but the effects were greater in high-walkability areas. Residents living in a highly walkable neighbourhood with one or more small- or medium-sized parks reported a 29% higher likelihood of being active during leisure time (OR: 1.29, 95%CI: 1.21–1.37) and a 48% greater likelihood of actively engaging in leisure and transportation-related physical activity (OR:1.48, 95% CI:1.38–1.57) compared to those who were living in areas with low walkability and no park access (referent) (Figure 1). In contrast, high-walkability neighbourhoods with no small or medium parks had a 16% increase in LTPA only. Associations were more modest in low-walkability neighbourhoods, where the addition of one or more parks of any size (low walkability/moderate to high park access) was associated with an increase in LPA and LTPA of only 4–7%. Similar patterns were observed with respect to the presence or absence of large parks within a community. As an exception, high walkability was associated with a ~40% increase in total physical activity (leisure and transportation combined) with no further benefit conferred by the presence of one or more large parks.

**Figure 2:**
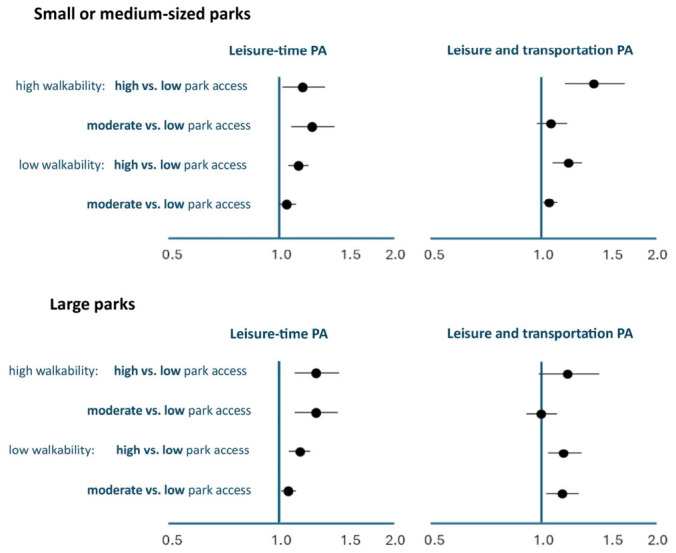
High and moderate park access verses low access within 800 m and the odds of being physically active, by park size and neighbourhood walkability. PA: physical activity; reference group: low park access; adjusted for age, sex, ethnicity, season, household income, neighbourhood walkability, and park access; analyses were stratified by walkability. High walkability: quintile 5, low walkability: quintiles 1–4. High park access was defined as ≥3 parks within 800 m, moderate park access was defined as 1–2 parks within 800 m, and low park access was defined as 0 parks within 800 m. Data sources: Data for LTA, LTPA and obesity were attained from Canadian Community Household Survey (CCHS) cycles 2007–2008, 2009–2010, 2011–2012, and 2013–2014; data for parks were attained from DMTI, Ministry of Education, and City of Toronto.

These findings appeared to be largely driven by areas that had very high access to parks, defined as three or more parks within 800 m (Figure 2). In highly walkable neighbourhoods, very high access to small- and medium-sized parks was associated with a 16% increase in LPA and a 27% increase in LTPA compared to high walkability alone (i.e., no park access), while very high access to large parks was associated with a 36% increase in LPA and a nonsignificant increase (18%) in LTPA. A similar pattern was noted in lower walkability neighbourhoods although the magnitude of these effects was diminished; very high access to parks of any size was associated with only a 13 to 16% increase in rates of LPA and LTPA. These findings appeared to be dose-related, as having only moderate access to parks (1–2 locations within 800 m) was generally associated with a smaller increase in physical activity levels of any type. Overall, residents living in high-walkability neighbourhoods engaged in greater amounts of leisure and leisure- and transportation-related physical activity compared with those living in low-walkability neighbourhoods. The benefits of high walkability on LTPA appeared to be augmented in the presence of small or medium parks.

### 3.2. Park Access, Walkability and Obesity

**Figure 3:**
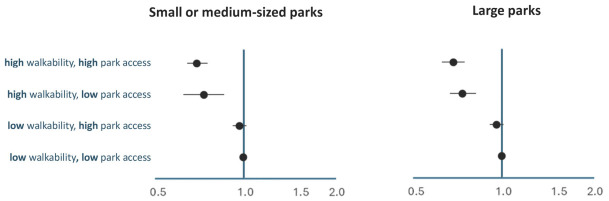
Combined effects of neighbourhood walkability and park access on the odds of obesity, by park size. Reference group: low walkability and low park access; adjusted for age, sex, ethnicity, season, household income, neighbourhood walkability, park access, and interaction terms between neighbourhood walkability and park access; high walkability was based on quintile 5 and low walkability was based on quintiles 1–4. High park access was defined as ≥1 parks within 800 m; low park access was defined as 0 parks within 800 m. Data sources: Data for LTA, LTPA and obesity were attained from Canadian Community Household Survey (CCHS) cycles 2007–2008, 2009–2010, 2011–2012, and 2013–2014; data for parks were attained from DMTI, Ministry of Education, and City of Toronto.

Figure 3 demonstrates the associations between park access, walkability and obesity. As demonstrated in the Figure, high walkability (with or without park access) was associated with the greatest reduction in the odds of obesity compared to communities with low walkability and no park access); with no or little additional benefit conferred by having one or more parks within walking distance. However, within highly walkable neighbourhoods, having three or more large parks within walking distance was associated with a further reduction in the odds of obesity (OR: 0.72 (0.55–0.94) compared to highly walkable areas without any access to parks (Figure S1). Very high access to small- or medium-sized parks was not associated with obesity rates in highly walkable areas. In low-walkability neighbourhoods, obesity rates were also unaffected by park access.

### 3.3. Sensitivity Analyses

Findings were relatively consistent in both younger (aged 20–49 yrs) and older adults (aged ≥ 50 yrs). Adults aged 20–49 years living in the most favourable environments (high walkability/moderate to high park access) reported higher levels of LPA and LTPA and lower levels of obesity, regardless of park sizes (Table S1). Similar but more modest trends were observed for older adults. Sensitivity analyses also confirmed a threshold effect between high walkability (Q5) and lower levels of walkability (Q1–4) with respect to physical activity (Figure S2) and obesity (Figure S3).

## 4. Discussion

Our findings suggest that neighbourhood parks have a strong influence on physical activity levels and obesity in the population. Among adults of all ages, those living within a short walking distance of local parks and greenspace had a greater likelihood of being physically active during their leisure time or during leisure and transportation-related activities combined. These findings are consistent with prior studies demonstrating positive associations between greenspace and physical activity. While these benefits were observed in a variety of urban settings, they appear to be even greater in neighbourhoods that are highly walkable, highlighting the importance of designing urban areas where these resources can be reached on foot. In contrast, the absence of a meaningful association between park access and physical activity in low-walkability areas suggests that proximity alone may be insufficient to support park use. In such environments, limited pedestrian connectivity and greater reliance on car-based transportation may reduce the likelihood of accessing nearby parks, even when they are geographically close. In addition, compositional differences in the populations living in different neighbourhoods, including their socioeconomic conditions, time constraints, and preferences for recreational activity, may further influence how residents engage with available greenspace. Nonetheless, access to parks appeared to confer additional benefits to those already living in a highly walkable area, with apparent increases in both leisure and transportation-related physical activity.

In recent decades, there has been a growing body of research illustrating the health benefits of parks and greenspace. Beyond their effects on physical activity, local parks and greenspace promote social cohesion and offer protection against excess heat, noise and air pollution [6,7,11,44]. As a co-benefit, spending time in nature appears to have favourable effects on mental health, sleep quality, cognitive function, stress, and loneliness [3,7,35,45]. Studies examining the association between neighbourhood greenspace and physical activity have been largely positive and the patterns we observed are similar to those seen in other studies from Australia, Europe, the United States, Canada, and Central America [3,9,20,46]. However, the literature overall has shown mixed results. Heterogeneity in the evidence base appears to be due in part to the use of variable methods [7,9,17,19,35]. This includes differences across studies regarding how park and greenspace access is measured (e.g., distance to parks or greenspace, travel time to the closest park, tree canopy coverage and/or quality of green space), how health outcomes are collected (i.e., self-report versus objective measures, individual vs. population data sources), and which population is being studied (i.e., children, young versus older adults) [7,9]. Furthermore, inadequately addressing important interactions between neighbourhood features, such as greenspace and walkability, likely also contributed to differences in findings across built environment studies [19,35].

A number of studies have found positive associations between greenspace and obesity [3,47]. In contrast, our study found that neighbourhood walkability, but not parks, had a significant impact on the odds of obesity. However, highly walkable neighbourhoods were also far more likely to offer access to parks. To date, very few studies have explored the combined effects of park access and neighbourhood walkability on physical activity and obesity. While it is often difficult to disentangle these effects, it is possible that some benefits previously attributed to park exposure were in fact due to neighbourhood walkability or other characteristics. Highly walkable neighbourhoods provide residents with an opportunity to engage in active transportation and to reduce one’s reliance on cars for mobility [19,48,49]. From previous studies, high neighbourhood walkability was associated with a reduced likelihood of being overweight or obese and of developing prediabetes, diabetes, hypertension and cardiovascular disease [24,25,26,27,28,29,30]. From our study, park access was associated with physical activity but generally offered little additional benefit with respect to obesity. An exception was in highly walkable neighbourhoods, where proximity to high access (i.e., three or more) large-sized parks was associated with a further reduction in the rate of obesity in the population. However, some subgroup estimates were accompanied by wider confidence intervals, indicating reduced precision, and should be interpreted with caution. Since larger parks often have walking loops, trails, playgrounds, sports amenities, and open spaces for a variety of recreational activities, they may support individuals to be even more physically active [15,17,50]. Together, these findings suggest that the health benefits of parks are maximized when embedded within walkable neighbourhoods, rather than implemented as standalone features.

Our study may have missed favourable associations between greenspace and body weight for several reasons. This may help explain why park access was not consistently associated with obesity in our analyses, despite observed associations with physical activity. First, there are inherent limitations in using BMI as a surrogate for metabolic health. In both normal and overweight individuals, moderate levels of physical activity (such as achieved through brisk walking) may yield beneficial effects on visceral (abdominal) adiposity in the absence of significant changes in BMI. Visceral adiposity is more closely associated with insulin resistance and the risk of type 2 diabetes and related diseases [51]. Populations that have different ethnoracial backgrounds have different BMI thresholds that predict the presence of visceral adiposity and insulin resistance [42,43]. In fact, the presence of the latter is more common in non-white groups who have a lower BMI. Studies examining the association between greenspace and health often fail to account for socioeconomic status or ethnicity, which is one reason why residual confounding occurs in epidemiological studies [7]. In our study, residents living in higher income communities reported being more physically active and had lower rates of overweight and obesity. High income neighbourhoods tend to be greener and have more amenities within parks, a concept known as green gentrification. To account for this, we adjusted for household income in all of our analyses.

This study has some limitations that are important to acknowledge. Our study was cross-sectional and despite using several cycles of the CCHS to create our sample, exposures and outcomes were captured at a single point in time. Therefore, our findings cannot be used to infer causation. Next, our outcomes were based on self-reported information and participants may have had incomplete recall or may have exaggerated the amount of physical activity they engaged in. Additionally, people who are more physically active may choose to live near a park and therefore may be systematically different than those who do not live near these resources. Further, we did not account for other park characteristics in our analyses, such as the presence of amenities, walking trails, sufficient lighting or safety. These features may be more common in higher income neighbourhoods due to greater investments in these areas. Although we adjusted for many factors in our analyses, including age, sex, season, household income, and self-reported ethnicity, unmeasured population characteristics may have resulted in residual confounding. Several prior studies used propensity score methods to account for systematic imbalances between populations living in high versus low walkability settings; however, these analyses yielded similar results as multivariate regression models alone [28]. Thus, it is unlikely that the use of more complex methods would have altered our findings. In addition, park data were obtained for a single year (2009) and therefore there is a potential for misclassification of exposure status over time. However, parks represent relatively stable features of the built environment, and substantial changes in park location or size over the study period are unlikely. Furthermore, residential self-selection may also influence observed associations, as individuals who value physical activity may preferentially choose to live in more walkable, park-rich neighbourhoods [52,53,54]. However, prior work suggests that adjustment for self-selection using propensity score methods does not materially alter associations between walkability and health outcomes. Nonetheless, residual confounding cannot be excluded. An additional limitation relates to the study period (2007–2014), which was chosen based on data availability when the study was conceptualized. This period predates an uptake in the use of digital technology and screen-based activities. Global estimates suggest that sedentary time has increased alongside widespread adoption of digital devices, potentially displacing time spent in outdoor or recreational activities [13,55]. As such, the relationships observed in this study may differ in more contemporary contexts. While accounting for such secular changes was beyond the scope of this research, future studies should examine how evolving patterns of technology use interact with built environment features to influence health behaviours. Finally, although we adjusted for household income, educational attainment was not included in the final models to avoid potential overadjustment. Education represents a distinct dimension of socioeconomic status, and residual confounding by SES may therefore remain.

Our study also has many strengths. We used data from 15 municipalities in Southern Ontario, Canada, which included both urban and suburban landscapes. Therefore, we had a sufficiently large sample size to assess the interaction between neighbourhood features. Ours was among the first large population-based studies to examine this issue and extends that of prior Canadian work using household survey and administrative data to understand the relationships between parks and greenspace and their impact on physical activity and obesity [20]. Our study contributes to this prior research by formally testing interaction effects between walkability and park access, while also examining dose–response relationships based on park count and size. Further, although, there is great heterogeneity in the evidence base on greenspace access and health, our study contributes to this growing field by supporting the notion that creating highly walkable environments with access to parks and greenspace is beneficial for promoting healthy living—by providing residents with more opportunities to engage in leisure and transportation-related physical activity to reduce their risk of obesity.

## 5. Conclusions

These findings have important implications for future research and policy development to address these global public health challenges through healthy and sustainable urban designs. According to the World Obesity Federation, an estimated 2.7 billion people are overweight or obese [51]. Physical inactivity is an important contributor to these trends and an essential target for public health intervention [13]. Our study revealed an important interplay between features that promote physical activity. In highly walkable neighbourhoods, the presence of small/medium and large parks was associated with a further increase in transportation-related and leisure-time physical activity, respectively. However, urban parks and greenspace may be insufficient in overcoming the inherent barriers imposed by unsupportive environments, such as those caused by low walkability. Understanding these multilayered and intersecting effects is a necessary step in designing neighbourhoods that optimally promote and sustain healthy behaviours, like physical activity. For instance, a key finding that may inform future planning is that the addition of a park in low-walkability areas may have limited impact. In contrast, walkable neighbourhoods appear to benefit most from greater park availability and size. Together, these findings suggest that planning should prioritize combined investments in both walkability and park access, rather than relying on single-component approaches alone. These findings remain relevant for contemporary urban planning and are reflected in the concept of a “15 minute city”, which promotes access to parks within walking distance of a resident’s home [23]. Regardless, our observation that neighbourhood features which promote physical activity differ across communities calls for more context-specific rather than a one-size-fits-all solution to address physical inactivity. With respect to parks and greenspace, it is essential to understand how local residents use these spaces and what attributes are essential to meet a community’s needs [56,57,58]. There is a move towards more inclusive mechanisms to incorporate community perspectives in greenspace planning. These processes may be essential to ensure that future policy efforts are both equitable and optimally effective in promoting physical activity and healthy living.

## Figures and Tables

**Table 1:** Baseline characteristics of study population by park access of any size within 800 m in Southern Ontario, Canada.

Variables	Any Park Access in 800 m	No Park Access in 800 m	Total Population	Standardized Difference *
Population size	34,075	7870	41,945	
Age (mean ± SD)	51.5 ± 18.4	50.1 ± 18.1	51.2 ± 18.3	0.080
Age groups				
*20–49*	46.8	50.3	47.5	0.071
*50+*	53.2	49.7	52.5	0.071
Sex (female)	56.6	56.6	56.6	
Ethnicity				
*White*	72.2	67.2	71.3	0.110
*Non-white*	23.3	28.1	24.4	0.118
Household income				
*Lowest income*	6.3	5.0	6.0	0.054
*Lower middle income*	15.1	13.3	14.8	0.051
*Upper middle income*	28.8	27.4	28.6	0.032
*Highest income*	42.2	46.7	43.1	0.090
Education				
*Less than high school (sec school)*	11.7	10.6	11.5	0.034
*Secondary or post-sec school*	22.4	22.5	22.4	0.003
*Post-secondary school*	63.1	64.4	63.3	0.028
Marital status				
*Married*	46.4	54.7	47.9	0.167
*Common-law*	5.2	4.2	5.0	0.045
*Widowed/Divorced/Separated*	22.5	18.6	21.7	0.096
*Single*	25.8	22.3	25.1	0.080
Walkability Quintile (Q)				
*Q1 (lowest)*	18.7	44.2	23.5	0.571
*Q2*	18.8	19.5	18.9	0.019
*Q3*	20.6	21.5	20.8	0.022
*Q4*	21.5	8.9	19.1	0.356
*Q5 (highest)*	20.3	5.8	17.6	0.443
Season				
*Summer*	33.6	35.1	33.9	0.030
*Temperate*	31.7	30.7	31.5	0.022
*Winter*	34.6	34.2	34.5	0.009

Data sources: Canadian Community Household Survey (CCHS) cycles 2007–2008, 2009–2010, 2011–2012, and 2013–2014; data for parks were attained from the 2009 CanMap Parks and Recreational polygon data for Southern Ontario. * Standardized differences are reported to compare baseline characteristics between groups with any park access within 800 m and no park access within 800 m. All values are percentages until otherwise indicated.

## Data Availability

The dataset from this study is held securely in coded form at ICES. While legal data sharing agreements between ICES and data providers (e.g., healthcare organizations and government) prohibit ICES from making the dataset publicly available, access may be granted to those who meet pre-specified criteria for confidential access, available at www.ices.on.ca/DAS (accessed on 21 February 2026; email: das@ices.on.ca). The full dataset creation plan and underlying analytic code are available from the authors upon request, understanding that the computer programs may rely upon coding templates or macros that are unique to ICES and are therefore either inaccessible or may require modification.

## Supplementary Materials

The following supporting information can be downloaded at: https://www.mdpi.com/article/10.3390/ijerph23060787/s1; Figure S1: Association between park access and the odds of obesity, by park size and neighborhood walkability; Figure S2: Walkability quintile and the odds of being physically active, by high/low access to small/medium- (A) or large-sized parks (B); Figure S3: Walkability quintile and the odds of obesity, by high/low access to small/medium- (A) or large-sized parks (B); Table S1: Combined effects of neighbourhood walkability and park access on the odds of being physically active, by park size and age group.
