# Supplementary material for: Association of Park Size, Access and Neighbourhood Walkability with Physical Activity and Obesity: A Cross-Sectional Analysis
Source: Int J Environ Res Public Health. 2026 Jun 11;23(6):787. doi: 10.3390/ijerph23060787 (PMC13299377; doi:10.3390/ijerph23060787)
Supplement: Supplementary file 1 [file ijerph-23-00787-s001.zip › ijerph-4226592-supplementary.pdf]

**Figure S1.** Association between park access and the **odds of obesity**, by park size and neighborhood walkability.

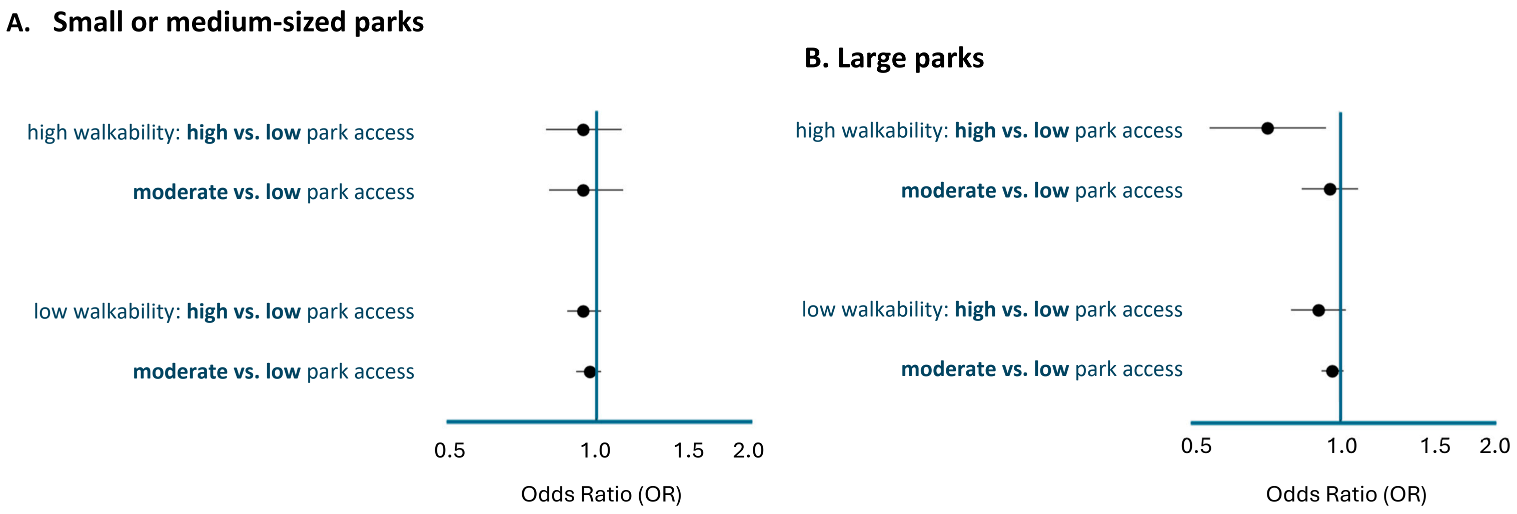


Referent: Low park access

Adjusted for age, sex, ethnicity, season, and household income, neighbourhood walkability, and park access

Analyses were stratified by walkability. High walkability was based on quintile 5 and low walkability was based on quintiles 1-4.

High park access was defined as ≥ 3 parks within 800m, moderate park access was defined as 1-2 parks within 800m, low park access defined as 0 parks within 800 m.

Data sources: Data for LTA, LTPA and obesity were attained from Canadian Community Household Survey (CCHS) cycles 2007-2008, 2009-2010, 2011-2012, and 2013-2014; data for parks were attained from DMTI, Ministry of Education, and City of Toronto.

**Figure S2.** Walkability quintile and the **odds of being physically active**, by high/low access to small/medium- (A) or large-sized parks (B).

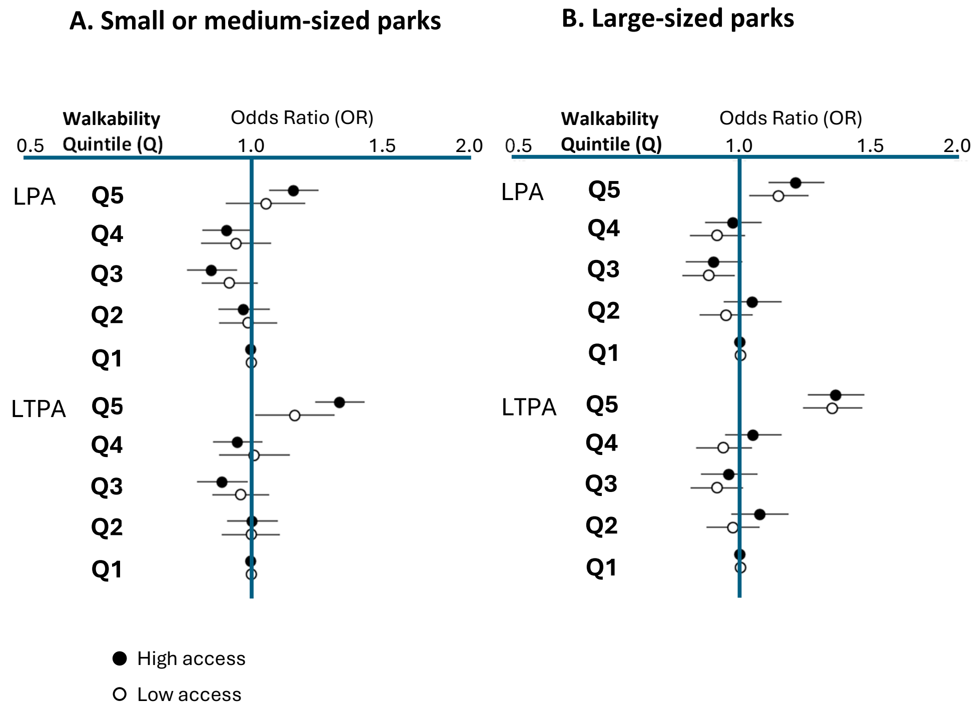


Referent: Lowest walkabiblity quintile (Q1)

Adjusted for age, sex, ethnicity, season, and household income, neighbourhood walkability, and park access

Analyses were stratified by high or low access to parks

High park access was defined as ≥ 1 parks within 800m, Low park access was defined as 0 parks within 800m

Data sources: Data for LTA, LTPA and obesity were attained from Canadian Community Household Survey (CCHS) cycles 2007-2008, 2009-2010, 2011-2012, and 2013-2014; data for parks were attained from DMTI, Ministry of Education, and City of Toronto.

**Figure S3.** Walkability quintile and the **odds of obesity**, by high/low access to small/medium- (A) or large-sized parks (B).

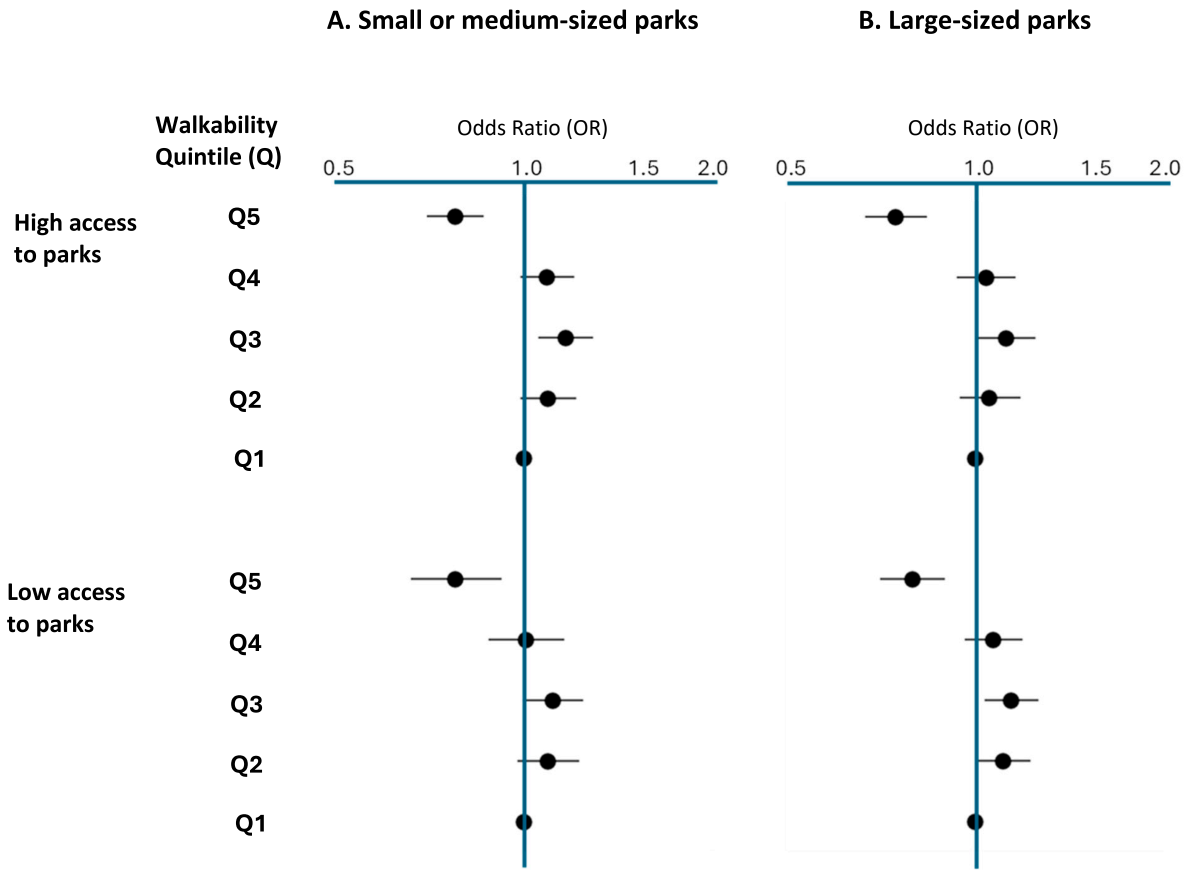


Referent: Lowest walkabiblity quintile (Q1)

Adjusted for age, sex, ethnicity, season, and household income, neighbourhood walkability, and park access

Analyses were stratified by high or low access to parks

High park access was defined as ≥ 1 parks within 800m, Low park access was defined as 0 parks within 800m

Data sources: Data for LTA, LTPA and obesity were attained from Canadian Community Household Survey (CCHS) cycles 2007-2008, 2009-2010, 2011-2012, and 2013-2014; data for parks were attained from DMTI, Ministry of Education, and City of Toronto.

**Table S1.** Combined effects of neighbourhood walkability and park access on the odds of being physically active, by park size and **age group.**

| | **Leisure Physical Activity** | **Leisure and Transportation Physical Activity** | **Obesity** |
|---|---|---|---|
| | Adjusted OR (95% CI) | Adjusted OR (95% CI) | Adjusted OR (95% CI) |
| ***Age 20-49*** | | | |
| **Small or medium parks** | | | |
| High walkability, high park access | 1.30 (1.19-1.42) | 1.61 (1.47-1.76) | 0.62 (0.55-0.70) |
| High walkability, low park access | 1.10 (0.93-1.31) | 1.21 (1.02-1.44) | 0.62 (0.49-0.79) |
| Low walkability, high park access | 1.06 (0.99-1.13) | 1.08 (1.02-1.16) | 0.93 (0.86-1.00) |
| Low walkability, low park access | | Referent | |
| **Large parks** | | | |
| High walkability, high park access | 1.30 (1.18-1.43) | 1.51 (1.37-1.67) | 0.66 (0.58-0.75) |
| High walkability, low park access | 1.19 (1.06-1.33) | 1.50 (1.33-1.68) | 0.64 (0.55-0.75) |
| Low walkability, high park access | 1.06 (0.99-1.13) | 1.07 (1.00-1.14) | 1.00 (0.93-1.08) |
| Low walkability, low park access | | Referent | |
| ***Age 50-74*** | | | |
| **Small or medium parks** | | | |
| High walkability, high park access | 1.29 (1.18-1.41) | 1.37 (1.25-1.49) | 0.77 (0.69-0.86) |
| High walkability, low park access | 1.07 (0.90-1.27) | 1.13 (0.96-1.34) | 0.84 (0.68-1.03) |
| Low walkability, high park access | 1.06 (0.99-1.12) | 1.06 (1.00-1.13) | 1.01 (0.94-1.08) |
| Low walkability, low park access | | Referent | |
| **Large parks** | | | |
| High walkability, high park access | 1.25 (1.14-1.38) | 1.32 (1.20-1.45) | 0.70 (0.62-0.79) |
| High walkability, low park access | 1.19 (1.06-1.33) | 1.28 (1.14-1.44) | 0.82 (0.71-0.95) |
| Low walkability, high park access | 1.03 (0.97-1.09) | 1.04 (0.98-1.10) | 0.92 (0.86-0.99) |

| | |
|---|---|
| Low walkability, low park access | Referent |

High walkability was based on quintile 5 and low walkability was based on quintiles 1-4.

High park access was defined as ≥ 1 parks within 800m, Low park access was defined as 0 parks within 800m.
